# Cytogenetic analysis of *Astylus antis* (Perty, 1830) (Coleoptera, Melyridae): Karyotype, heterochromatin and location of ribosomal genes

**DOI:** 10.1590/S1415-47572010005000050

**Published:** 2010-06-01

**Authors:** Ernani de Oliveira Mendes-Neto, Marcelo Ricardo Vicari, Carlos Campaner, Viviane Nogaroto, Roberto Ferreira Artoni, Mara Cristina Almeida

**Affiliations:** 1 Departamento Genética e Evolução, Universidade Federal de São Carlos, São Carlos, SPBrazil; 2 Departamento Biologia Estrutural, Molecular e Genética, Universidade Estadual de Ponta Grossa, Ponta Grossa, PRBrazil; 3 Museu de Zoologia, Universidade de São Paulo, São Paulo, SPBrazil

**Keywords:** sex chromosomes, meiosis, metaphase, FISH, 18S rDNA

## Abstract

Cytogenetic analysis of *Astylus antis* using mitotic and meiotic cells was performed to characterize the haploid and diploid numbers, sex determination system, chromosome morphology, constitutive heterochromatin distribution pattern and chromosomes carrying nucleolus organizer regions (NORs). Analysis of spermatogonial metaphase cells revealed the diploid number 2n = 18, with mostly metacentric chromosomes. Metaphase I cells exhibited 2n = 8II+Xyp and a parachute configuration of the sex chromosomes. Spermatogonial metaphase cells submitted to C-banding showed the presence of small dots of constitutive heterochromatin in the centromeric regions of nearly all the autosomes and on the short arm of the X chromosome (Xp), as well as an additional band on one of the arms of pair 1. Mitotic cells submitted to double staining with base-specific fluorochromes (DAPI-CMA_3_ ) revealed no regions rich in A+T or G+C sequences. Analysis of spermatogonial mitotic cells after sequential Giemsa/AgNO _3_ staining did not reveal any specific mark on the chromosomes. Meiotic metaphase I cells stained with silver nitrate revealed a strong impregnation associated to the sex chromosomes, and *in situ* hybridization with an 18S rDNA probe showed ribosomal cistrons in an autosomal bivalent.

## Introduction

The suborder Polyphaga is the most numerous of the order Coleoptera and displays the greatest structural and biological diversity of species (Gillot, 1995; Costa, 1999). The superfamily Cleroidea belongs to the suborder Polyphaga and comprises approximately 10,000 taxonomically described species (Costa, 2003), distributed among eight families (Lawrence and Newton, 1995), such as Melyridae, with approximately 5000 species, 68 of which occur in the Neotropical region (Costa, 2003). The genus *Astylus* belongs to the family Melyridae and includes a number of Brazilian species, such as *Astylus antis*, *A. quadrilineatus*, *A. sexmaculatus* and *A. variegatus*, which are best known for visiting the flowers of cultivated plants, such as corn, cotton and sorghum (Rosseto and Rosseto, 1976; Souza and Carvalho, 1994; Ventura *et al.*, 2007).

In the superfamily Cleroidea, only 16 species have been cytogenetically analyzed, 12 species belonging to the family Cleridae and four species of the family Melyridae. The 12 species of Cleridae, distributed among five genera (*Enoclerus*, *Priocera*, *Thanasimus*, *Trichodes* and *Necrobia*), exhibit karyotype uniformity, *i.e.*, 2n = 18, the basal sex determination system for Coleoptera, Xyp, and meta/submetacentric morphology for all chromosomes (Smith, 1953; Virkki, 1963; Smith and Virkki, 1978; Yadav and Dange, 1989; Schneider *et al.*, 2007a). However, the four species belonging to the family Melyridae display differences both in their chromosome number and their sex determination system (Smith and Virkki, 1978). Chromosome morphology was only described for *A. variegates*, in which all chromosomes are metacentric (Schneider *et al.*, 2007a).

Cytogenetic data using differential staining in species of Cleroidea are limited to *A. variegatus*, which has small blocks of heterochromatin in the pericentromeric region of all chromosomes, except Xyp. In this species, the nucleolus organizer region (NOR) is located in autosome pair 2 (Schneider *et al.*, 2007a).

In Coleoptera with the Xyp sex determination system, it is common to find nucleolus material associated to the sex chromosomes. Furthermore, there are a number of different mechanisms described to explain the association and segregation of these chromosomes in meiosis, depending on their degree of differentiation. These mechanisms oscillate between a nucleolus association and/or a synaptic association (Smith and Virkki, 1978; Juan *et al.*, 1993; Petitpierre, 1996). In Coleoptera, however, NORs are located in either autosomes and/or sex chromosomes (Almeida *et al.*, 2000; Schneider *et al.*, 2007a, 2007b). The few studies that have employed fluorescent *in situ* hybridization (FISH) in Coleoptera have found conflicting results between the location of the rDNA genes and the silver staining, particularly regarding the sex chromosomes of the Xyp system in some species. Therefore, the nucleolus theory for the maintenance and segregation of the sex chromosomes belonging to this system (Weber, 1971; Drets *et al.*, 1983; Virkki, 1983; Postiglioni and Brum-Zorilla, 1988; Postiglioni *et al.*, 1991; Juan *et al.*, 1993; Maffei *et al.*, 2001) has been questioned (Juan *et al.*, 1993; Moura *et al.*, 2003; Schneider *et al.*, 2007a, 2007b).

In order to understand how chromosome evolution occurred in the different species of this group, it is important to establish the constitutive heterochromatin distribution pattern and to identify the NOR-bearing chromosomes. These were the aims of the present study, in addition to chromosomally characterize the species *Astylus antis*, using both the mitotic karyotype and meiotic cells.

## Materials and Methods

The 17 specimens of *Astylus antis* (Perty, 1830) analyzed were collected in the cities of Carambeí (S 24°58'071”; W 50°06'817”) and Ponta Grossa (S 25°08'985”; W 49°58'992”) in the region of Campos Gerais, Paraná, Brazil.

Cytological preparations were obtained from the gonads of adult male individuals. The gonads were removed in insect saline solution, treated with hypotonic solution (tap water) for six minutes and fixed in Carnoy I. Then, the gonads were macerated in 45% acetic acid solution, and the slides were dried on a metal plate at a temperature of 35 to 40 °C; later on, the slides were stained with 3% Giemsa in pH 6.8 phosphate buffer for 15 min.

The C-banding and base-specific fluorochrome staining (DAPI/CMA_3_) methods described by Sumner (1972) and Schweizer (1980), respectively, were used to determine the distribution and the AT/GC content of the constitutive heterochromatin. The silver nitrate impregnation method described by Howell and Black (1980) and the fluorescent *in situ* hybridization (FISH) method with 18S rDNA described by Pinkel *et al.* (1986) were used to identify the chromosomes bearing NORs. The partial 18S rDNA probe (732 pb) was obtained through amplification by PCR labeled with biotin-14-dATP hapten (Invitrogen), using the cloned 18S fragment of *Omophoita octoguttata* (Coleoptera) as template. The hybridization signals were detected using avidin-fluorescein isothiocyanate (Avidin-FITC, Sigma). For amplification of the signals, we used anti-avidin biotinylated (Sigma) and Avidin-FITC (Sigma) conjugated antibodies. Overall hybridization was performed under high stringency conditions (2.5 ng/μL probes, 50% deionized formamide, 10% dextran sulfate, 2XSSC at 37 °C overnight). After hybridization, the slides were washed in 15% formamide/0.2XSSC at 42 °C for 20 min, 0.1XSSC at 60 °C for 15 min, and 4XSSC /0.05% Tween at room temperature for 10 min, the latter consisting of two washes of 5 min each. Chromosomes were counterstained with DAPI (0.2 mg/mL) in anti-fade solution.

Approximately 40 cells from each specimen were examined. Chromosomes were counted and identified whenever possible. The best mitotic and meiotic cells in both conventional and differential staining were photographed under an optical photomicroscope (Olympus BX41), with a 100x immersion objective. The metaphase cells submitted to the base-specific fluorochromes and FISH were photographed with a digital camera (Olympus C-5060 5.1 Megapixel) with specific filters, or recorded by real-time digital imaging with a DP-71 camera and DP controller software.

The karyotypes were arranged and numbered in decreasing order, based on size and morphology of the chromosomes, and the homologous chromosomes were tentatively paired to facilitate presentation and comparison, as proposed by Levan *et al.* (1964).

## Results

###  Conventional staining

**Figure 1:**
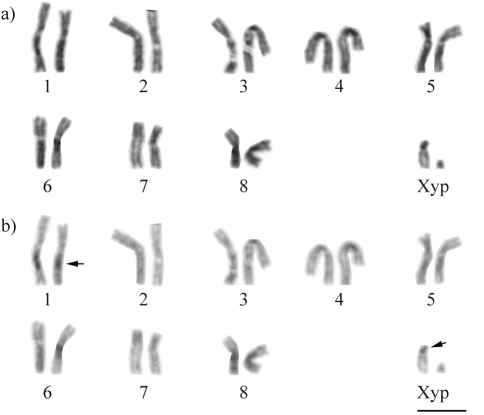
Mitotic karyotype of a male *Astylus antis* specimen with 2n=18=16+X+yp: a. chromosomes stained with Giemsa; b. the same cell after C-banding, showing the centromeric heterochromatin region on the X chromosome (larger arrows) and an additional band on one of the arms of pair 1 (smaller arrow). Bar = 5 μm.

Analysis of spermatogonial metaphase cells revealed the diploid chromosome complement 2n = 18 = 16+Xyp. Most of the autosomes were metacentric, only pairs 5 and 7 were submetacentric. The Xp chromosome was submetacentric, and the yp chromosome was extremely small, which made it impossible to determine its morphology but it may be acrocentric (Figure 1a).

**Figure 2:**
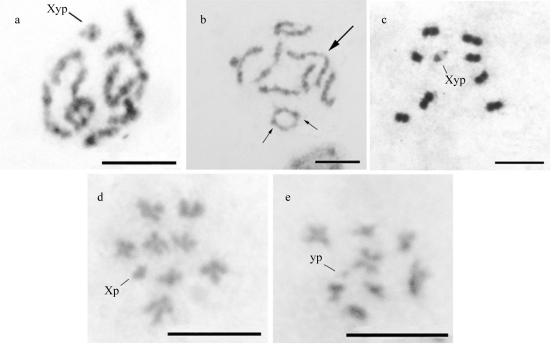
Meiotic cells from *Astylus antis:* a. pachytene cells; b. diplotene with 2n = 8II+Xyp (arrows = chiasmata); c. metaphase I cell, showing 2n = 8II+Xyp; d. and e. metaphase II cells, with n = 8+X and n = 8+y, respectively. Bar = 5 μm.

The pachytene cells showed all bivalents completely, including the sex chromosomes, which displayed a parachute configuration. Small positive heteropycnotic blocks were found in these cells (Figure 2a). The study of diplotene cells revealed the occurrence of one or two chiasmata per bivalent (Figure 2b). The metaphase I cells examined showed the chromosome meioformula 2n = 8II+Xyp and the parachute configuration of the sex chromosomes (Figure 2c). The metaphase I cells showed a haploid complement n = 8+Xp or n = 8+yp (Figures 2d and 2e), indicating normal chromosome segregation during anaphase I. The yp chromosome exhibited negative heteropycnosis in the majority of meiotic phases analyzed.

###  Differential staining

C-banding and base-specific fluorochrome (DAPI/CMA_3_) staining of spermatogonial metaphase cells revealed the presence of small dots of heterochromatin in the centromeric regions of most autosomes, as well as an interstitial band on one of the arms of the pair 1 chromosomes and on the short arm of the Xp chromosome (Figure 1b), but with no differentiation between AT- and GC-rich sites (data not shown). Sequential Giemsa/AgNO_3_ staining of spermatogonial mitotic cells revealed no NOR-specific labeling on the chromosomes.

**Figure 3:**
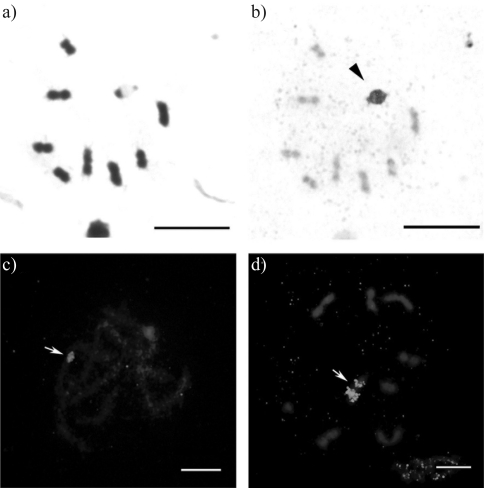
Meiotic cells from *Astylus antis*: a. metaphase I cell stained with Giemsa, 2n = 8II+Xyp; b. the same cell stained with silver nitrate, showing strong impregnation associated to the sex chromosomes (arrowhead); c. and d. pachytene and metaphase I cells, respectively, hybridized with an 18S rDNA probe, showing an autosomal bivalent with a fluorescent signal (arrows). Bar = 5 μm.

Meiotic cells submitted to C-banding and base-specific fluorochrome staining showed no specific AT- or GC-rich blocks or sites. Sequential Giemsa/AgNO_3_ staining of meiotic metaphase I cells revealed strong silver nitrate impregnation on the sex chromosomes (Figures 3a and 3b). This block was interpreted as argyrophilic material. FISH analysis of pachytene and metaphase I cells using an 18S rDNA probe revealed a fluorescent signal strongly associated to an autosomal bivalent and no labeling on the sex chromosomes (Figures 3c and 3d).

## Discussion

The results obtained for *Astylus antis* regarding the sex determination system, chromosome morphology, C-band distribution pattern and location of NORs are in agreement with those described for many other species of Coleoptera. The 2n = 18 = 16+Xyp chromosome number is similar to that described for the 12 species of Cleridae (Smith, 1953; Virkki, 1963; Smith and Virkki, 1978; Yadav and Dange, 1989), whereas it is in agreement with only one of the four Melyridae species analyzed - *A. variegatus* (Schneider *et al.*, 2007a). Furthermore, the chromosome formula 2n = 18 differs from the 2n = 20 = 18+Xyp described as basal for the order by Smith (1950) and supported by a number of recent studies (Maffei *et al.*, 2000; 2001; Moura *et al.*, 2003; Rozek *et al.*, 2004; Almeida *et al.*, 2000; Schneider *et al.*, 2007a, 2007b). The difference between the chromosome number found and the basal number may be explained by the occurrence of fusion-type chromosome rearrangements between two pairs of autosomes, followed by pericentric inversion, as also proposed for *A. variegatus* by Schneider *et al.* (2007a).

According to Smith and Virkki (1978), the evolutionary tendency for Coleoptera of the suborder Polyphaga was to maintain the chromosome number close to the original, whereas, for representatives of Adephaga, the tendency was to increase the chromosome number through autosomal centric fission. However, centric fusion events appear to be less frequent, as there are only few species with low chromosome numbers.

In the few cytogenetically studied species from the family Cleridae, no change in sex chromosomes was observed, so the Xyp sex determination system was maintained. Nevertheless, in the family Melyridae, maintenance of the Xyp system has been observed in *Hoppigiana hudsonica* (2n = 6II+Xyp) and *A. variegatus* (2n = 8II+Xyp), while *Collops* sp (2n = 8II+X0) and *Endeodes collaris* (2n = 9II+X0) lost the yp chromosome, giving rise to an X0 sex determination system (Smith, 1953; Virkki, 1963; Smith and Virkki, 1978; Yadav and Dange, 1989; Schneider *et al.*, 2007a).

Small karyotype differences were found when comparing the results from *A. antis* with the description for *A. variegatus* regarding the metacentric morphology of all the chromosomes, the behavior of the sex chromosomes and the presence of B chromosomes (Schneider *et al.*, 2007a). Thus, it can be inferred that small rearrangements of the inversion type and differentiation of the sex chromosomes occurred during the chromosome differentiation of these species.

Negative heteropycnosis as observed in the yp chromosome of *A. antis* has also been found in some species of Coleoptera, including *A. variegatus*. Differences involving heteropycnosis may occur due to differential chromosome condensation and/or the presence of a special type of chromatin (Virkki, 1967; Yadav *et al.*, 1985; Almeida *et al.*, 2000). The number of chiasmata found in *A. antis* is in agreement with the number described for most species of Coleoptera, as well as for *Enoclerus* sp, *Necrobia ruficollis* and *Astylus variegatus*, which belong to the superfamily Cleroidea (Virkki, 1963; Yadav and Dange, 1989; Schneider *et al.*, 2007a).

The centromeric constitutive heterochromatin pattern observed in *A. antis* by C-banding is in line with that described for various species of Coleoptera, including those with the Xyp sex determination system, such as *Epilachna paenulata* (Drets *et al.*, 1983), *Gonocephalum patruele*, *G. rusticum*, *Hegeter grancanariensis*, *Pachychila sublunata*, *Tenebrio molitor*, *Tentyria grossa* (Juan and Petitpierre, 1989), *Epicauta atomaria*, *Palembus dermestoides* (Almeida *et al.*, 2000), *Eriopis connexa* (Maffei *et al.*, 2000), *Phyllophaga (Phytalus) vestita* (Moura *et al.*, 2003), *Adelocera murina*, *Oedemera podagraridae*, *O. virescens* (Rozek *et al.*, 2004), and *Astylus variegatus* (Schneider *et al.*, 2007a). However, the interstitial band found on the long arm of the chromosomes of pair 1 in *Astylus antis* is not present in the karyotype of *A. variegatus*.

From the results obtained with the double staining (DAPI/CMA_3_), which coincide with those obtained by C-banding, it can be concluded that the weakly fluorescent signals are heterochromatic regions, but with no differentiation between AT- and GC-rich sites. A number of studies have attempted to explain the conflicting results between the content of DNA bases and responses to base-specific fluorochromes (Comings and Drets, 1976; Saitoh and Laemmli, 1994; Vicari *et al.*, 2008). The fluorochrome DAPI binds to DNA, but its fluorescence is significantly enhanced in AT-rich domains. According to Comings and Drets (1976), Comings (1978) and Johnston *et al.* (1978), the antibiotic daunomycin only emits fluorescence when the AT content exceeds 65%. According to Vicari *et al.* (2008), the absence of fluorescence on large heterochromatic blocks of the fish *Astyanax janeiroensis* is due to the effect of competition between two families of repetitive DNA co-located in the same chromosome domains. Contrarily, competition and/or excitation energy transference between DAPI and CMA_3_, together with the absence of AT/GC differentiation in these regions (Zimmer *et al.*, 1971) and the state of heterochromatic compaction, could explain the coinciding results of the C-bands and the DAPI-CMA_3_ fluorochrome signals (Saitoh and Laemmli, 1994).

Moura *et al.* (2003) obtained similar results using triple CMA_3_/DA/DAPI staining: they found that in *Phyllophaga (Phytalus) vestita* there was no difference between the CMA_3_ and DAPI signals, both of which were positive. *Lyogenys fuscus* displays a strong fluorescent signal by DAPI in the pericentromeric region of all chromosomes. Likewise, Vitturi *et al.* (1999) found that positive CMA_3_ regions coinciding with C bands were also DAPI-positive in *Thorectes intermedius* (Geotrupidae).

Analyzing *Epilachna paenulata* with the fluorochrome method (Quinacrine HCl or Hoechst 33258), Drets *et al.* (1983) found intensely fluorescent regions in the centromeric region of the autosomes, a region rich in AT sequences. Juan *et al.* (1991) studied *Tenebrio molitor* and Plohl *et al.* (1993) analyzed testicular cells from *Tribolium confusum* using DA/DAPI fluorochromes and found that the pericentromeric and centromeric regions of all chromosomes in the complement were rich in AT sequences.

FISH with the 18S rDNA probe revealed a fluorescence signal strongly associated to an autosomal bivalent in *Astylus antis*. The labeling obtained by silver nitrate staining on the sex chromosomes without the presence of ribosomal cistrons (which were detected by FISH only in one autosomal bivalent) in *A. antis* is in agreement with the results obtained for other species of Coleoptera with the Xyp system (Vitturi *et al.*, 1999; Colomba *et al.*, 2000a; Moura *et al.*, 2003; Bione *et al.*, 2005). It is also in agreement with a survey carried out by Schneider *et al.* (2007b), in which 81% of Adephaga and Polyphaga species had NORs located on the autosome pairs. The silver staining of these nonspecific blocks may result from the presence of an argyrophilic substance, which theoretically facilitates the configuration, maintenance and segregation of the sex chromosomes of the Xyp system, as described by a number of authors (Virkki *et al.*, 1990, 1991; Juan *et al.*, 1993; Petitpierre, 1996; Moura *et al.*, 2003; Bione *et al.*, 2005; Schneider *et al.*, 2007a, 2007b). A large number of studies have associated these nonspecific silver nitrate marks with argyrophilic proteins and heterochromatic regions, particularly proteins associated to these regions (Virkki *et al.*, 1991; Vitturi *et al.*, 1999; Colomba *et al.*, 2000a, 2000b, 2004, 2006; Bione *et al.*, 2005).

The karyotype differences observed regarding chromosome morphology, C-banding patterns and behavior of the sex chromosomes in meiosis of *A. antis* in comparison to the description of *A. variegatus* suggest that the karyotype evolution of these two species may have involved different types of chromosome rearrangements, such as small inversions and the addition of heterochromatin. Regarding the plesiomorphic characteristics for the order, the reduction in number may have occurred due to pericentric inversion, followed by fusion between autosomes, with no involvement of the sex chromosomes.
